# Exome-Wide Rare Variant Analysis From the DiscovEHR Study Identifies Novel Candidate Predisposition Genes for Endometrial Cancer

**DOI:** 10.3389/fonc.2019.00574

**Published:** 2019-07-05

**Authors:** Manu Shivakumar, Jason E. Miller, Venkata Ramesh Dasari, Radhika Gogoi, Dokyoon Kim

**Affiliations:** ^1^Biomedical and Translational Informatics Institute, Geisinger, Danville, PA, United States; ^2^Department of Genetics, Perelman School of Medicine, University of Pennsylvania, Philadelphia, PA, United States; ^3^Weis Center for Research, Geisinger Clinic, Danville, PA, United States; ^4^Department of Biostatistics, Epidemiology and Informatics, Perelman School of Medicine, University of Pennsylvania, Philadelphia, PA, United States; ^5^Institute for Biomedical Informatics, University of Pennsylvania, Philadelphia, PA, United States

**Keywords:** endometrial cancer, rare variant, cancer predisposition gene, EHR, EHR-linked Biobank, DiscovEHR cohort

## Abstract

Endometrial cancer is the fourth most commonly diagnosed cancer in women. Family history is a known risk factor for endometrial cancer. The incidence of endometrial cancer in a first-degree relative elevates the relative risk to range between 1.3 and 2.8. It is unclear to what extent or what other novel germline variants are at play in endometrial cancer. We aim to address this question by utilizing whole exome sequencing as a means to identify novel, rare variant associations between exonic regions and endometrial cancer. The MyCode community health initiative is an excellent resource for this study with germline whole exome data for 60,000 patients available in the first phase, and further 30,000 patients independently sequenced in the second phase as part of DiscovEHR study. We conducted exome-wide rare variant association using 472 cases and 4,110 controls in 60,000 patients (discovery cohort); and 261 cases and 1,531 controls from 30,000 patients (replication cohort). After binning rare germline variants into genes, case-control association tests performed using Optimal Unified Approach for Rare-Variant Association, SKAT-O. Seven genes, including *RBM12, NDUFB6, ATP6V1A, RECK, SLC35E1, RFX3* (*Bonferroni-corrected P* < 0.05) and *ATP8A1* (suggestive *P* < 10^−5^), and one long non-coding RNA, *DLGAP4-AS1* (*Bonferroni-corrected P* < 0.05), were associated with endometrial cancer. Notably, *RECK*, and *ATP8A1* were replicated from the replication cohort (suggestive threshold *P* < 0.05). Additionally, a pathway-based rare variant analysis, using pathogenic and likely pathogenic variants, identified two significant pathways, pyrimidine metabolism and protein processing in the endoplasmic reticulum (*Bonferroni-corrected P* < 0.05). In conclusion, our results using the single-source electronic health records (EHR) linked to genomic data highlights candidate genes and pathways associated with endometrial cancer and indicates rare variants involvement in endometrial cancer predisposition, which could help in personalized prognosis and also further our understanding of its genetic etiology.

## Introduction

MyCode Community Health initiative is a precision medicine initiative by Geisinger Health System (1). As part of the initiative, blood and other samples are collected from the patients who have consented to participate in the MyCode Community Health initiative. The samples are stored in a systemwide biobank and are sequenced at Regeneron Genetics Center as part of the DiscovEHR project. The high-throughput sequencing data coupled with longitudinal electronic health records (EHR) has been used for genetic research. The genetic data from DiscovEHR has been successfully used to detect various disease-causing variants, which confer increased risk to develop one or more of 21 conditions including hereditary breast and ovarian cancer, familial hypercholesterolemia, cardiomyopathy, Marfan syndrome, and Lynch syndrome (2). The clinically actionable pathogenic variants identified are delivered to the patients through the return of results program at Geisinger Health System and their EHR is updated with the information, which can be readily accessed by their provider (1). As of February, 2019, the results for 1,048 MyCode participants who have one or more of clinically actionable conditions were delivered through the return of results program (2). One of the primary conditions diagnosed as part of the MyCode initiative is Lynch syndrome, and 99 patients out of 1,048 were diagnosed with Lynch Syndrome. Lynch syndrome is caused by germline mutations in one of several DNA mismatch repair genes. Among Lynch syndrome patients, endometrial cancer is the second most diagnosed condition (3). Women with Lynch syndrome have about a 50% chance of developing endometrial cancer (3, 4). Familial risk of endometrial cancer increased by ~2 fold with a first-degree female relative with endometrial cancer (5). However, the germline abnormalities associated with Lynch syndrome only explain a small fraction of heritability of endometrial cancer, which suggests there are other rare germline variants that can help explain the heritability of the disease.

The germline variants have been studied for finding associations with various diseases on a genome-wide scale. Many successful genome-wide association studies (GWAS) have identified common variants associated with multiple complex diseases, including cancer. The NHGRI-EBI catalog has almost 60,000 unique SNP-trait associations (6). Many GWAS have been also conducted on endometrial cancer. One of the recent meta-analysis studies using 7,737 endometrial cancer cases and 37,144 controls identified seven loci associated with endometrial cancer (7). However, to date, all the GWAS using common variants are only able to explain <6% of the familial relative risk for endometrial cancer (7–10). Since common variants discovered to be associated with endometrial cancer have only modest effect size, the missing heritability could be further explained by rare variants.

Rare variants are known to play an essential role in human diseases. Due to the evolutionary purifying selection, deleterious alleles are likely to be rare (11). Moreover, many rare variant analysis studies have found variants to be associated with various cancer types, such as prostate cancer (12), melanoma (13), colorectal cancer (14), urinary tract cancer (15), etc. Notably, rare pathogenic germline variants in various cancers have been linked to functional consequences (16). To the best of our knowledge, rare variants have not been widely studied in endometrial cancer. An exome-wide rare variant association study was conducted in 2014, but they could not find any variants significantly associated with endometrial cancer (17). Therefore, there is a need to further investigate rare variants that are responsible for the endometrial cancer susceptibility. Identifying cancer predisposition genes based on pathogenic rare variants could give novel insight into the genetic basis of endometrial cancer and could be valuable for preventive markers and precision medicine.

In summary, we set out to discover rare variants associated with endometrial cancer using whole exome data where 472 endometrial cancer patients and 4,110 non-cancer patients were pulled from a single hospital system. Their phenotypes and demographics were derived from their EHRs stored in system-wide EPIC database. The rare variants were binned into genes and pathways, and subsequently, statistical tests were run to test the association of biologically-informed units with endometrial cancer. Moreover, the significant results from the discovery phase were validated using an independent DiscovEHR replication cohort with 261 endometrial cancer patients and 1,531 non-cancer patients, also pulled from the same hospital system.

## Results

### Study Design and Quality Control

Whole exome sequencing was performed on 60,000 samples as part of DiscovEHR study. From 60,000 samples all patients with endometrial cancer (*N* = 481) and matched controls (*N* = 4,403) were selected. Further, an independent cohort of 30,000 samples was also sequenced as part of DiscovEHR study, and endometrial cancer (*N* = 263) and controls (*N* = 1,586) was pulled as a replication dataset. The controls consisted of women with no diagnosis of cancer and were retrieved by matching the age and body mass index (BMI) with all cancer patients in the cancer registry. Age and BMI were calculated as described in the Methods section. For case population, all patients diagnosed with any type of cancer were retrieved from the cancer registry and then a subset of patients with international classification of diseases for oncology (ICD-O) site codes relevant to endometrial cancer, specifically C54.0: Isthmus uteri, C54.1: Endometrium, C54.3: Fundus uteri, and C54.9: Corpus uteri were selected.

**Figure 1:**
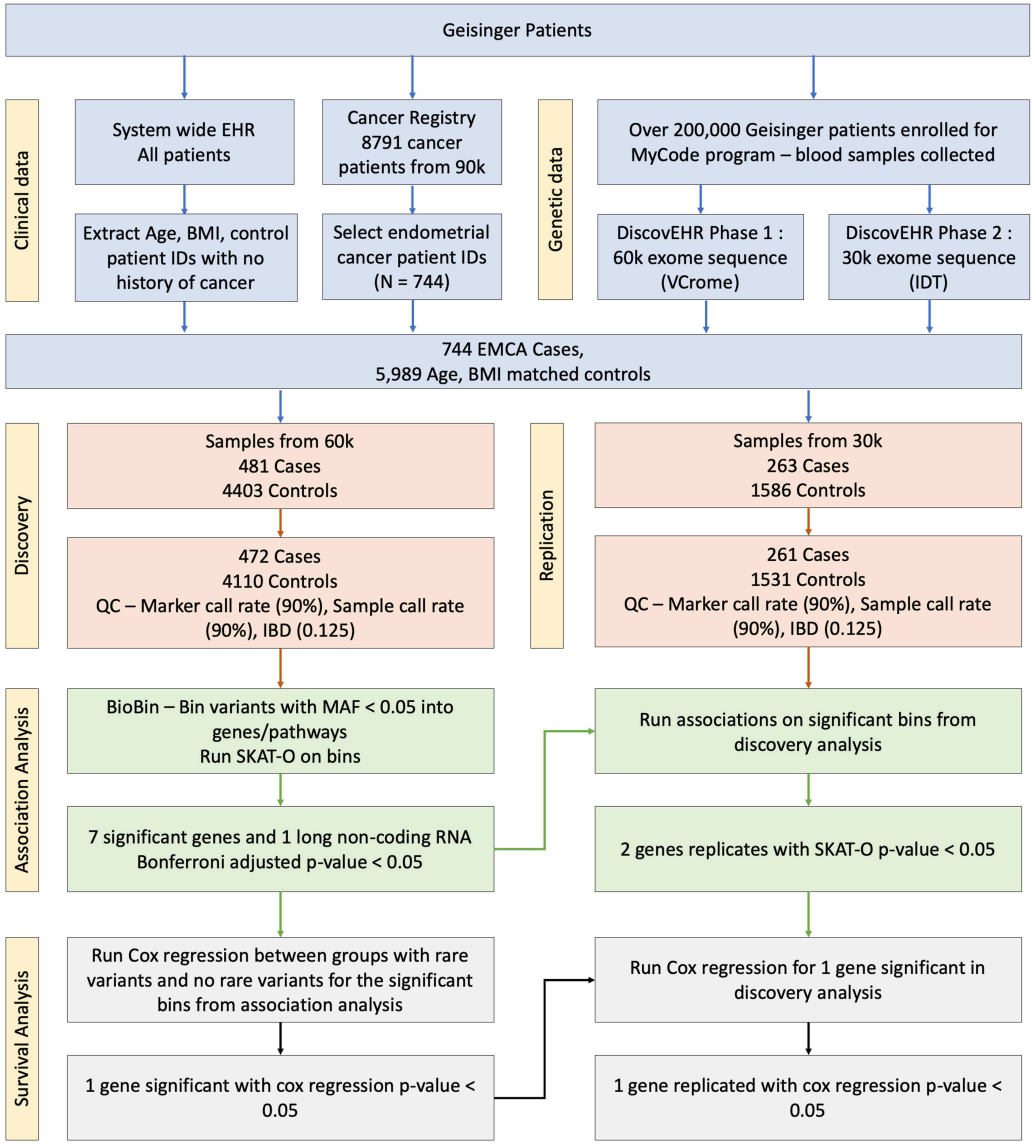
Schematic overview of the association study. The blood samples were collected and sequenced as part of MyCode and DiscovEHR projects. The phenotype information was pulled from the cancer registry and EHR.

After retrieving the patient IDs for case and control populations, their whole exome sequencing data was retrieved from DiscovEHR. Further, quality filters were applied to the data before performing a rare variant analysis. Any loci with <90% call rate in the exome data were removed, and all patients were confirmed to have genotype call rate >90%. All third-degree relatives (Identity by descent (IBD) > 0.125) were removed. Additionally, principal components were calculated to adjust for population stratification using EIGENSOFT (18). There were 299 related patients with IBD <0.125 with 9 cases and 290 controls in discovery dataset and 55 related patients with two cases and 53 controls in replication dataset that were removed. Further, 10 controls from discovery and replication datasets were removed because of missing BMI in their EHR. After the marker quality control (QC) step, the whole exome sequence had 2,431,845 rare variants with minor allele frequency (MAF) <0.05, and the rare variants were binned into gene bins determined by Entrez Gene annotations. The schematic overview of the study is shown in Figure 1.

The population characteristics for case and control populations after QC steps are summarized in Table 1. The average age of endometrial cancer patients was 60 years with a standard deviation of 11.69 years and control population had an average age of 58 with a standard deviation of 14.45. Further, in case population 64 were deceased, and 408 were still alive as of May 2017, when the data was retrieved from the cancer registry. The control population had 222 deceased and 3,888 patients alive. The table also provides the American Joint Committee on Cancer (AJCC) stage information and the number of patients with a history of any cancer in the family. The two tailed *t*-test statistics for age and BMI between case and control group in discovery and replication are listed in Supplementary Table 1.

**Table 1:** Characteristics for the study population.

**Characteristic features**	**Discovery EMCA cases**	**Replication EMCA cases**	**Discovery controls**	**Replication controls**
Number of patients	472	261	4,110	1,531
**AGE**				
Average	60.12	60.97	58.36	56.45
Median	60.00	61.00	59.00	57.00
Standard deviation	11.69	10.51	14.45	14.05
**BMI**				
Average	37.83	36.56	31.73	31.72
Median	36.72	35.18	30.00	30.00
Standard deviation	9.55	10.10	8.12	8.07
**VITAL STATUS**				
Alive	408 (86.4%)	248 (95.0%)	3,888 (94.6%)	1,509 (98.6%)
Dead	64 (13.6%)	13 (5.0%)	222 (5.4%)	22 (1.4%)
**AJCC STAGE**				
Stage 1/2	252 (53.4%)	134 (51.3%)	Not applicable	Not applicable
Stage 3/4	36 (7.6%)	21 (8.0%)	Not applicable	Not applicable
Unknown	184 (40.0%)	106 (40.6%)	Not applicable	Not applicable
Family history of cancer	355 (75.2%)	205 (78.5%)	Not available	Not available

### Gene-Based Rare Variant Analysis

**Figure 2:**
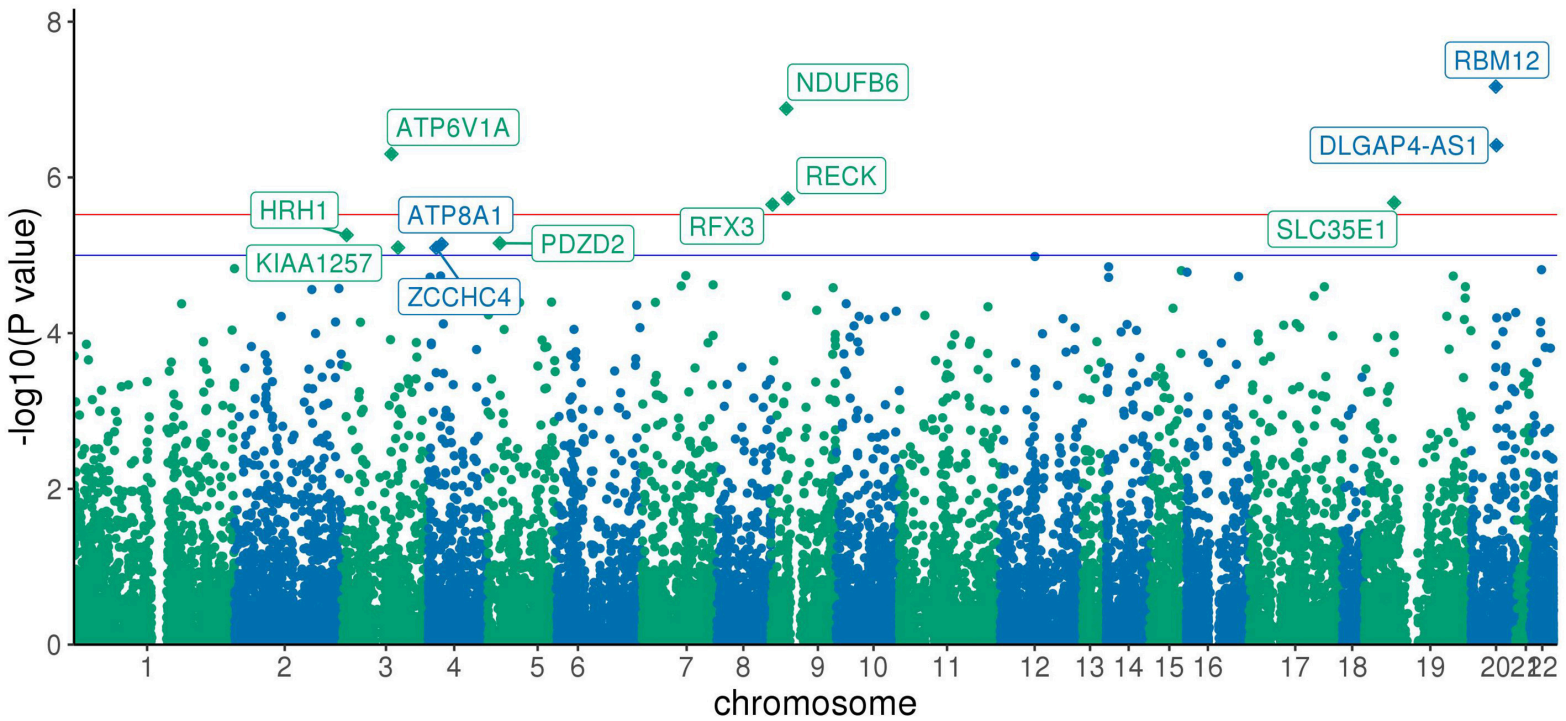
Manhattan Plot of SKAT-O association results: Each point represents one of the 20,385 genes plotted against x-axis being chromosome position and y-axis log transformed SKAT-O *p*-value. All the genes above the red line are Bonferroni significant, and genes above the blue line have a *p*-value < 1 × 10^−5^.

BioBin (19–21), a rare variant analysis tool, was used to bin rare variants into genes, and SKAT-O (22) was used to identify associations between genes and phenotype of interest. Any variant below MAF of 5% was considered rare. In the discovery analysis, 2,431,845 rare variant loci were binned into 22,126 genes. Further, any genes with <20 minor allele counts (MAC) were filtered out, leaving only 20,385 genes. Both burden and dispersion tests can be applied to test for association. However, SKAT-O is a unified approach that optimally combines the burden and non-burden sequence kernel association test (SKAT) and increases statistical power to detect associations (22). After running SKAT-O on 20,385 genes, the association *p*-values were further adjusted for multiple testing using Bonferroni correction and any adjusted *p*-value below 0.05 was considered significant. We identified six genes and one long non-coding RNA that reached global significance—*RBM12, NDUFB6, DLGAP4-AS1, ATP6V1A, RECK, SLC35E1*, and *RFX3* as shown in Manhattan plot in Figure 2.

The *RECK* gene was found to be associated with endometrial cancer and was replicated (*p*-value < 0.05). *ATP8A1* showed a suggestive association (*p*-value < 10^−5^) in the discovery analysis and was also replicated (*p*-value < 0.05). Table 2 shows the number of loci, allele counts, SKAT-O *p*-value, and Bonferroni corrected *p*-value of the six protein-coding genes, one long non-coding RNA as well as additional suggestive gene, *ATP8A1* from discovery and replication analysis. Table 2 also summarizes the distribution of MAC between case and control populations.

**Table 2:** Genes associated with endometrial cancer after Bonferroni correction in discovery analysis.

**Gene information**		**Discovery**					**Replication**			
**Gene**	**Chr: GRCh38 position**	**N locus**	**MAC case**	**MAC control**	**SKAT-O *p*-value**	**Bonferroni corrected *p*-value**	**N locus**	**MAC case**	**MAC control**	**SKAT-O *p*-value**
*RBM12*	20:35648925–35664956	74	34 (6.78%)	164 (3.89%)	6.82E-08	0.0014	64	77 (6.13%)	520 (4.77%)	0.5394
*NDUFB6*	9:32553526–32573184	111	86 (15.68%)	630 (14.08%)	1.31E-07	0.0027	23	12 (4.6%)	60 (3.92%)	0.9169
*DLGAP4-AS1*	20:36507702–36573275	98	37 (6.14%)	225 (4.87%)	3.86E-07	0.0081	49	33 (10.73%)	205 (11.1%)	0.8315
*ATP6V1A*	3:113747019–113812058	114	57 (10.38%)	362 (7.93%)	5.00E-07	0.0104	74	21 (7.66%)	183 (10.12%)	0.2754
***RECK***	9:36036905–36124455	188	158 (20.34%)	1127 (21.56%)	1.86E-06	0.0388	130	140 (27.59%)	1142 (34.16%)	**0.0450**
*SLC35E1*	19:16549837–16572382	87	79 (14.83%)	524 (12%)	2.11E-06	0.0441	54	37 (14.18%)	266 (16.2%)	0.4076
*RFX3*	9:3218297–3526529	176	178 (23.73%)	1279 (21.44%)	2.23E-06	0.0465	148	119 (31.42%)	855 (37.17%)	0.5394
ATP8A1^*^	4:42408373–42657105	268	302 (41.74%)	2553 (39.1%)	7.17E-06	0.1494	174	160 (37.55%)	908 (39.32%)	**0.0129**

*The associations were adjusted for age, BMI, and four principal components. The table shows the number of loci, allele counts in cases and controls and association results for discovery and replication datasets*.

*N locus, Total number of genomic loci binned in the gene; MAC Case, Total minor allele count in the gene in case population with the percentage samples that have rare variants; MAC Control, Total minor allele count in the gene in control population with the percentage samples that have rare variants*.

* *Suggestive association—not Bonferroni significant but p-value < 10^–5^ in discovery dataset and p-value < 0.05 in replication dataset*. *The two genes marked in bold were replicated with suggestive threshold P < 0.05*.

**Figure 3:**
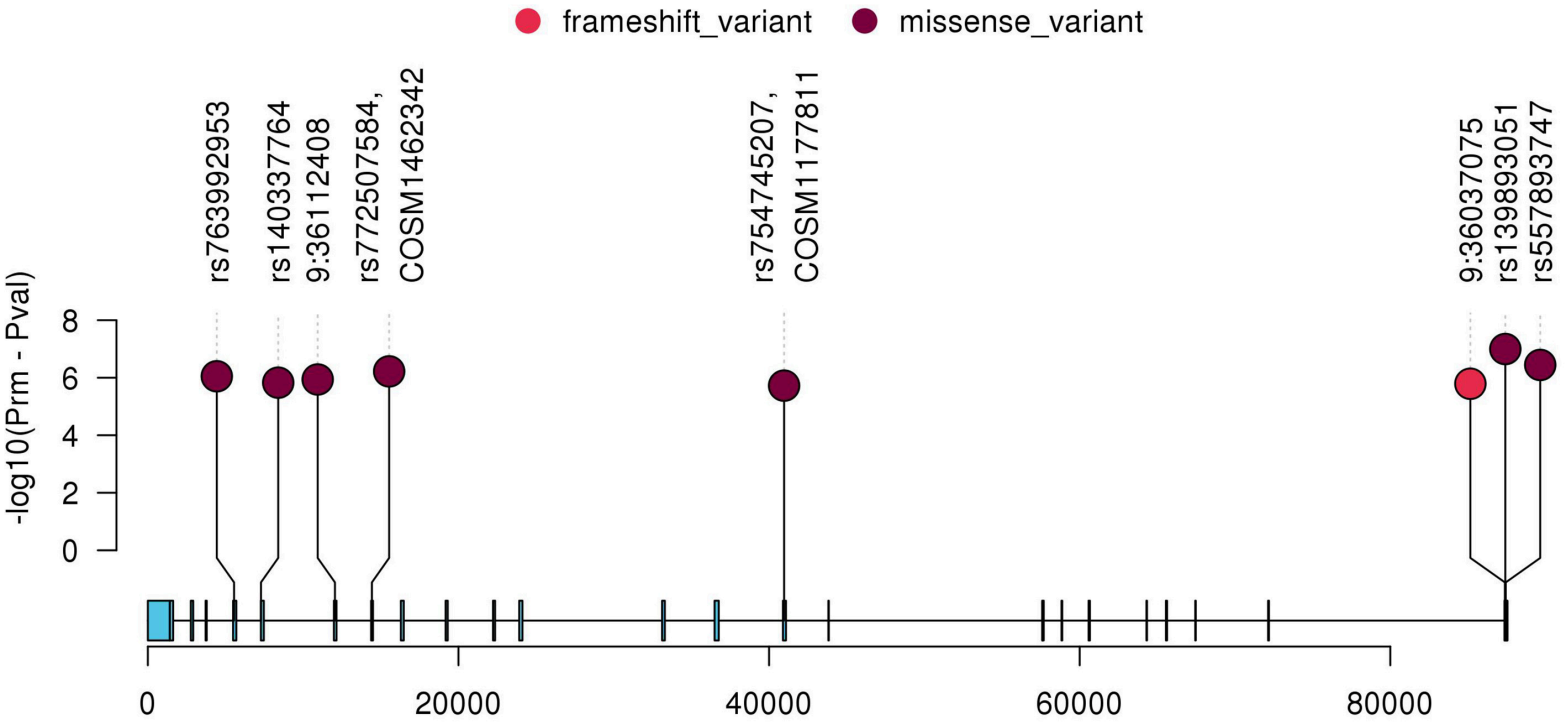
Plot of all variants with lower *P*_rm_ in *RECK* which were classified as moderate or high impact by VEP. The y-axis represents negative log scaled *P*_rm_-*P*_val_ where *P*_val_ is the original SKAT-O *p*-value listed in Table 2, and the x-axis is relative genomic coordinate in the gene.

To further characterize the contribution of each locus binned in the significant genes, the association tests were rerun by removing each locus at a time from the gene/bin and *p*-values (*P*_rm_) were generated. A significant increase in *P*_rm_ (less significant) would indicate the higher significance of the locus in the gene and no change in *P*_rm_ would indicate the locus in consideration is insignificant. After running the tests, the *P*_rm_ was lower for 245 loci out of 1,136 total loci binned. All the loci with positive *P*_rm_ in *RECK* and annotated as moderate or high impact by Variant Effect Predictor (VEP), are listed in Table 3 and the corresponding plot in Figure 3. A complete list of loci and their *P*_rm_ for all other significant genes can be found in Supplementary Tables 2–9.

**Table 3:** The variants with lower *P*_rm_ in *RECK*.

**Chr: GRCh38 Pos**	**RS_ID/COSMIC ID**	**Alt/ref**	**Aminoacids**	**MAF**	**Consequence**	**VEP impact**	***P*_**rm**_**
9:36083487	rs754745207&COSM1177811	A/G	A/T	0.00010912	missense_variant	MODERATE	3.15E-06
9:36037075	–	G/GGGGCCTGGCTC	GGLAP/GX	0.00010912	frameshift_variant	HIGH	2.98E-06
9:36117172	rs140337764	C/T	C/R	0.00021825	missense_variant	MODERATE	2.89E-06
9:36112408	–	A/T	H/Q	0.00021825	missense_variant	MODERATE	2.67E-06
9:36118904	rs763992953	A/G	E/K	0.00021825	missense_variant	MODERATE	2.50E-06
9:36110029	rs772507584&COSM1462342	C/G	R/P	0.00010912	missense_variant	MODERATE	2.30E-06
9:36037021	rs557893747	C/T	L/P	0.00054633	missense_variant	MODERATE	2.13E-06
9:36037047	rs139893051	A/G	A/T	0.00065517	missense_variant	MODERATE	1.94E-06

### Variant Annotation

**Figure 4:**
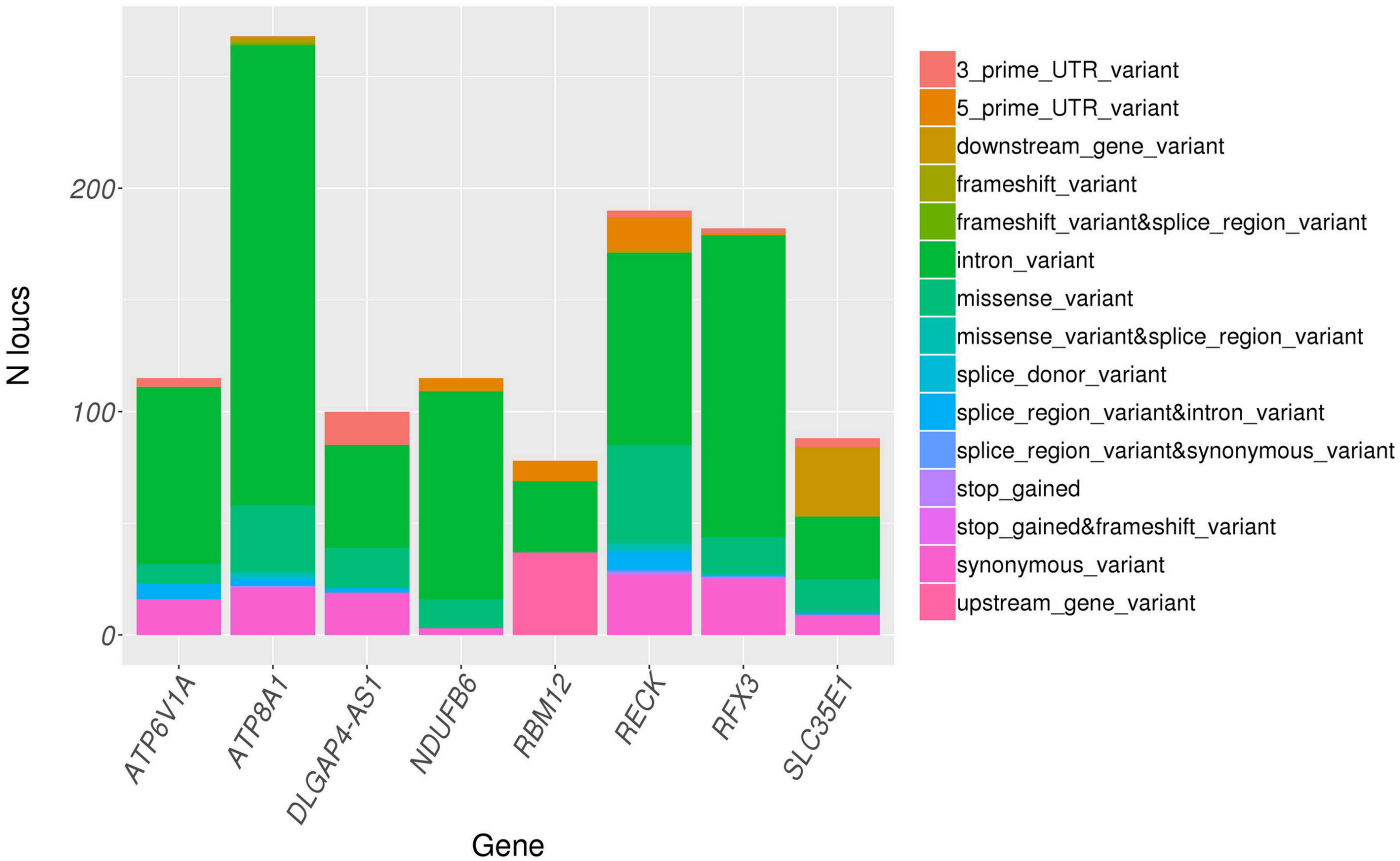
Distribution of variant consequence as determined by VEP for all rare variants in significant genes.

Variants in significant genes were annotated using ClinVar (2018-07) and Variant Effect Predictor (VEP v92) to determine the clinical significance, effect of variants on the protein and implications in human inherited diseases. ClinVar is a public archive that connects human variation to phenotypes, the clinical significance, relationship to human health, and other supporting data obtained through submissions by various groups and aggregated to reflect both consensus and conflicting assertions (23). VEP provides information about the variants' location, gene/transcript affected by variants, types of mutation (i.e., stop gained, missense, stop lost, and frameshift) and protein change scores, which indicate possible partial/complete loss of function of the protein due to amino acid substitution (24). None of the identified variants were found in ClinVar database. However, all variants were successfully annotated using VEP. The distribution of variant types across significant genes as annotated by VEP is shown in Figure 4. Majority of the variants across all significant genes are intron variants (62.0%), followed by missense (12.8%), synonymous (10.6%), and other variants (14.6%).

Identified variants were also annotated with a COSMIC database, which contains a manually curated list of somatically acquired mutation in various cancers. Out of 1,136 variants in the significant genes, 50 variants were also found in the COSMIC database (Supplementary Table 10). Three variants in *RBM12*, one variant in *NDUFB6*, ten variants in *DLGAP4-AS1*, five variants in *ATPV1A*, eleven variants in *RECK*, four variants in *SLC35E1*, eight variants in *RFX3*, and eight variants in *ATP8A1* were known somatically acquired mutations in various cancers. The subset of 50 variants with the COSMIC annotation that had lower *P*_rm_ is summarized in Table 4 with tissue type where the somatic mutations were observed. Two of the somatic mutations COSM1177811 in *RECK* and COSM1036559 in *ATP6V1A* were observed in Endometrium.

**Table 4:** The variants with lower *P*_rm_ found in COSMIC.

**Gene**	**Chr: GRCh38 Pos**	**rsid**	**COSMIC ids**	Tissue^*^
*DLGAP4-AS1*	20:36525992	rs971669684	COSM5601362, COSM5601361	Skin
*RBM12*	20:35652667	rs747020729	COSM5039294	Liver
*DLGAP4-AS1*	20:36548162	rs7273824	COSM3693464	Large intestine, prostate
*RFX3*	9:3247951	rs2229356	COSM3763880	Large intestine
*DLGAP4-AS1*	20:36526879	rs114982034	COSM4098029, COSM4098028	Stomach
*ATP6V1A*	3:113795187	rs771311957	COSM4583806, COSM1036559	Bone, **endometrium**
*ATP8A1*	4:42485617	rs370223580	COSM1184146, COSM1184145	Large intestine
*RECK*	9:36083487	rs754745207	COSM1177811	**Endometrium**
*SLC35E1*	19:16553901	rs773244448	COSM1391284, COSM1391283	Large intestine
*ATP8A1*	4:42586383		COSM3603994, COSM3603993	Skin
*RECK*	9:36110029	rs772507584	COSM1462342	Large intestine
*ATP8A1*	4:42443590	rs140420171	COSM1184148, COSM1184147	Large intestine, skin

*Somatic mutation in variant rs771311957 in ATP6V1A and variant rs754745207 in RECK were discovered in Endometrium*.

* *Primary tissue where the somatic mutations were found as cataloged by COSMIC database*.

*The tissue source where the somatic mutations were found in endometrium in COSMIC database are marked in bold*.

### Pathway-Based Rare Variant Analysis

Rare variants were also binned into pathways using KEGG pathway annotations. As pathway bins would include a large number of loci with a limited sample size, only loci categorized as pathogenic or likely pathogenic with at least one star in ClinVar and loci categorized as high impact by VEP were binned. The association analysis based on pathway bins was also performed using SKAT-O. Out of 317 pathways tested, six pathways were significant with *p*-value < 0.1 after FDR (Table 5) and two pathways—pyrimidine metabolism and protein processing in endoplasmic reticulum were found to be significant with *p*-value < 0.05 after Bonferroni correction. However, none of the pathways observed to be associated with endometrial cancer were replicated.

**Table 5:** Pathways associated with endometrial cancer in discover analysis.

**KEGG pathway**	***N* locus**	**MAC case**	**MAC control**	**SKAT-O *p*-value**	**FDR corrected *p-*value**	**Bonferroni corrected *p*-value**
Pyrimidine metabolism	271	173 (30.29%)	1384 (28.73%)	5.86E-06	0.0019	0.0018
Protein processing in endoplasmic reticulum	302	96 (18%)	654 (14.28%)	9.60E-05	0.0152	0.0304
Pentose and glucuronate interconversions	120	85 (16.95%)	670 (14.65%)	8.31E-04	0.0704	0.2635
Pancreatic secretion	293	85 (15.68%)	543 (12.04%)	1.18E-03	0.0704	0.3728
RNA polymerase	69	20 (4.023%)	152 (3.5%)	1.28E-03	0.0704	0.4044
Pantothenate and CoA biosynthesis	64	26 (5.51%)	164 (3.92%)	1.33E-03	0.0704	0.4223

*The associations were adjusted for age, BMI, and four principal components. The table shows the number of loci, allele counts in cases and controls and association results for discovery and replication datasets*.

*N locus, Total number of genomic loci binned in the gene; MAC Case, Total minor allele count in the gene in case population with the percentage samples that have rare variants; MAC Control, Total minor allele count in the gene in control population with the percentage samples that have rare variants*.

### Survival Analysis

**Figure 5:**
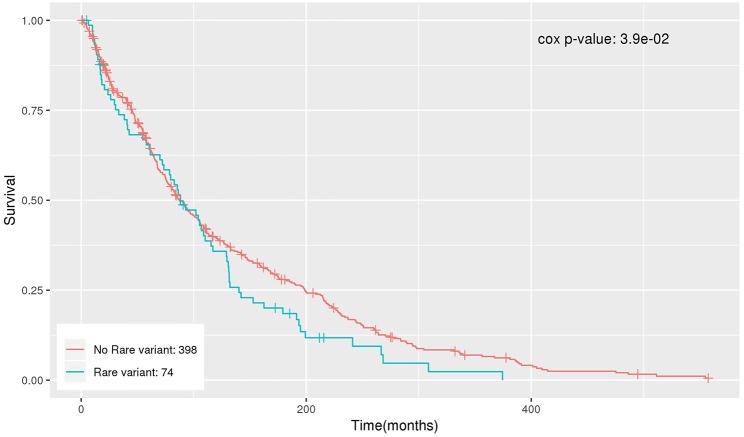
Kaplan–Meier plot for the survival of patients with rare variants in *NDUFB6*.

The significant genes were further analyzed to determine their association with survival outcome of endometrial cancer patients. The survival analysis was performed using Cox regression, adjusting for age and BMI. *NDUFB6* was observed to be significantly associated with survival with Cox regression *p*-value < 0.05 (Table 6). Out of 472 endometrial cancer patients, 74 had rare variants in *NDUFB6*, and they had a lower survival rate in comparison to endometrial cancer patients with no rare variants in *NDUFB6* (Figure 5).

**Table 6:** Cox regression results for gene *NDUFB6*.

**Dataset**	***N*_**rare**_**	***N*_**norare**_**	**Cox regression *p*-value**
Discovery	74 (15.7%)	398 (84.3%)	0.039
Replication	12 (4.6%)	249 (95.4%)	0.037

*N_rare_, number of patients with any rare variant in the gene; N_norare_, number of patients with no rare variant in the gene*.

## Discussion

The results from this study illustrate that a population from a single hospital system can be used to identify rare germline variants associated with endometrial cancer diagnosis. Genome-wide rare variant analysis using the DiscovEHR cohort identified seven genes and one long non-coding RNA to be associated with endometrial cancer, of which two genes *RECK* and *ATP8A1* were replicated (suggestive threshold *P* < 0.05). Additionally, the significance of the variants was evaluated by the backward elimination approach. Variants were also annotated using ClinVar and VEP to examine the variant consequence and any known associations. Further, survival analysis was run to access how the presence of rare variants in a gene influenced the survival of the patient. In summary, many rare variants were discovered, which positively contributed to the association of gene, and some of them were also found to be known somatic mutations in cancer including endometrial cancer. One of the genes found to be associated with endometrial cancer, *NDUFB6*, was also found to be significantly associated with survival.

Several genes identified in this study have been previously implicated in endometrial cancer or other cancers. The second most significant gene found to be associated with endometrial cancer in this study, *NDUFB6* is a nuclear-encoded subunit of NADH-ubiquinone oxidoreductase, also known as Complex I (CI), which is the largest complex of the electron transport chain in mitochondria. CI is known to play a role in tumorigenesis, resistance to cell death and metastasis (25). Moreover, various oncocytic cancer cells have an excessive number of mitochondria. Mutations in *NDUFB6* have been observed in oncocytic thyroid tumor (26) and downregulation of *NDUFB6* due to the loss in 9p24.1-p13.3 is known to be responsible for metastasis in renal cell carcinoma (27). Besides, in this study, there were 13 missense variants out of which 7 were predicted to be damaging by polyphen score that could disrupt the function of *NDUFB6*. Moreover, the other 99 variants in intron or UTR region could modify the gene expression. They also could result in alternative splicing of *NDUFB6* as there are three distinct isoforms that are encoded by the transcript variants (RefSeq, Jan 2011). Further, one of the endometrial cancer studies investigating mitochondrial DNA mutations conducted an immunohistochemistry analysis of various types of type 1 endometrial cancer samples by staining for nuclear-coded *NDUFB6* and mitochondria-coded *MTND6*. In a 0–4 intensity score for staining, samples with oncocytic-like foci showed about 23% (3/13) complete loss of staining, 23% (3/13) partial loss of staining with an intensity score of 2 and 23% (3/13) partial loss of staining with the intensity score of 3 for *NDUFB6* (28). However, in case of endometroid samples with no specific differentiation aspects, 92% (12/13) showed complete staining (intensity 4). Thus, the evidence strongly suggests a role of *NDUFB6* in oncocytic endometrial carcinoma and further studies would be required to elucidate precise mechanisms. The survival analysis performed in this study also showed endometrial cancer patients with rare variants in *NDUFB6* have a significantly lower survival rate than endometrial cancer patients with no rare variants reemphasizing its possible role in endometrial cancer. Another gene found to be associated with endometrial cancer in this study, *SLC35E1* is known to be upregulated in late-stage endometrial endometrioid carcinoma (29). It is also known to have differential membrane proteome expression between normal and inflammatory breast cancer cells, which is a rare and very aggressive form of breast cancer (29, 30). The *SLC35E1* is not well-studied, and its mechanism of action in endometrial cancer is not well-understood.

The disruption of tumor suppressor genes and activation of oncogenes are common in cancers. The gene *RECK*, which was replicated in this study, is known to be a tumor suppressor (31). *RECK* negatively regulates some matrix metalloproteinases which are known to facilitate tumor invasion and metastasis (31). The epigenetic downregulation of *RECK* is known to stimulate invasion and migration in colon cancer (32), breast cancer (33), prostate cancer (34), lung cancer (35), and gastric cancer (36). Moreover, *RECK* is also part of KEGG pathway “MicroRNAs in cancer.” *RECK* has already been suggested as a promising prognostic marker, and therapeutic agent in the cancers mentioned above (37) and potentially could apply to endometrial cancer. Altogether, 189 variants were discovered in *RECK*, of which stop gained (*N* = 1), frame shift (*N* = 1), and missense variants (*N* = 44) could lead to abnormal protein product and disrupt tumor suppressor function of gene *RECK*. Particularly, *RECK* is known to produce two proteins which have opposing effects, the shorter isoform of *RECK* leads to faster cell migration (38). Other variants in intron, splicing region and UTR region could also promote cancer by downregulating canonical *RECK* isoform or alternative splicing of *RECK*, producing the shorter isoform. Other candidate genes found to be associated with endometrial cancer, *ATP6V1A* and *ATP8A1* act as oncogenes. *ATP6V1A* is known to drive proliferation and invasion in gastric cancer (39, 40) and *ATP8A1* in non-small cell lung cancer (41). The most significantly associated gene in this study, *RBM12* is associated with colorectal cancer (42) and tumorigenesis of Meibomian cell carcinoma (42, 43).

One of the pathways discovered by binning rare variants into KEGG pathways, Protein processing in the endoplasmic reticulum is associated with endometrial cancer (44, 45). Disruption in protein processing in endoplasmic reticulum could cause endoplasmic stress and activation of the unfolded protein response (UPR) and GRP78 which facilitates growth and invasion of endometrial cancer (44, 45). Another pathway, Pyrimidine metabolism was also significantly associated with endometrial cancer. Pyrimidines include cytosine, thymine, and uracil which are the basic building blocks of DNA and RNA. Disorders of purine and pyrimidine metabolism are known to increase cancer risk and even act as tumor suppressor depending upon the type and site of alterations (46).

Although we found significant associations, further studies are required to elucidate the functional and molecular mechanisms of the variants and genes in endometrial cancer. A potential limitation of our study is the replication cohort was not sufficiently powered to replicate results with genome-wide significance. Even though we found genome-wide significant genes associated with endometrial cancer in discovery dataset, the results need to be further confirmed by other independent studies, which is common practice for GWAS. Moreover, bigger sample sizes would increase statistical power and help us detect more rare variants and genes with modest effects. DiscovEHR study is ongoing, and participants/patients are still being enrolled and sequenced. Future studies could be conducted to replicate the results when more sequence data is available. Another limitation is that our data consists predominantly of patients with European ancestry due to the inherent ethnic distribution of Geisinger patients. Thus, we can only discover variants associated with European ethnicity. That being said, association analyses in a homogeneous population can be more powerful because the pool of case and controls are not divided across populations, and it can result in more robust associations. This limitation may be addressed in the future as DiscovEHR has started recruiting MyCode participants from geographical areas with a diverse ethnic population.

In conclusion, we have identified seven genes and one long non-coding RNA that are associated with endometrial cancer. At least two of the genes found have some known role in endometrial cancer. Additionally, many genes are associated with other cancers. We suggest that the genes and variants we identified in this study could help explain a fraction of the endometrial cancer heritability, facilitate personalized prognosis, and also aid in increasing our understanding of endometrial cancer etiology.

## Methods

### Study Population

Geisinger Health System is a health care provider in south-central and northeastern Pennsylvania and southern New Jersey. All the patients who use Geisinger health services are eligible to participate in MyCode community initiative and the study population consisted of these participants. As part of MyCode, all patients who enroll in the program are sequenced regardless of the medical conditions they have. The blood samples were collected at Geisinger and sequenced by Regeneron Genetics Center as part of DiscovEHR study. To date, whole exome sequencing has been performed on approximately 90,000 samples and are linked to their EHR under a protocol approved by Geisinger Institutional Review Board. Additionally, Geisinger also maintains a separate cancer registry, which contains information on all patients who have been diagnosed with cancer. The Geisinger cancer registry also contributes patient data to the National Cancer Database. There were 8,791 patients out of 90,000 sequenced patients diagnosed with any type of cancer from the cancer registry and 481 EMCA cases were identified among them using ICD-9 site codes—C54.3, C54.9, C54.1, and C54.0. The control population consisted of a subset of age and BMI matched patients out of 90,000 sequenced samples with no history of cancer diagnosis based on the absence of any ICD9/ICD10 code related to cancer in a problem-list entry of the diagnosis code, an inpatient hospitalization-discharge diagnosis code, or an encounter diagnosis code. The age for cancer patients was taken as age at initial diagnosis of cancer and for controls, the current age or age at death depending on whether they are alive or dead, respectively. BMI was calculated using median BMI for a year from initial diagnosis for cases. The BMI for controls were calculated using median BMI for a year from the current date for controls still alive and median BMI for a year from date of death for controls who were dead.

### Sample Preparation, Sequencing, and Quality Control

The sample preparation is described in detail in Dewey et al. (47). The DNA samples were transferred using 2D matrix tubes (Thermo Scientific) logged in LIMS (Sapio Sciences) and stored in automated biobank at −80°C (LiCONiC Tubestore). The sample quality was tested by running 100 ng of sample on a 2% pre-cast agarose gel (Life Technologies). Additionally, the quantity of sample was determined by fluorescence. The exome capture was prepared through a fully automated approach developed at Regeneron using a custom reagent kit from Kapa Biosystems. The captured DNA was PCR amplified and quantified by qRT—PCR (Kapa Biosystems).

The Exome Sequencing was performed at Regeneron Genetics Center. The 60,000 samples from phase 1 were sequenced using NimbleGen probe target-capture (SeqCap VCRome), and 30,000 samples from phase 2 were sequenced using a slightly modified version of xGen capture (Integrated DNA Technologies), which had supplemental probes added to capture regions of the genome well-covered by VCRome capture reagent but poorly covered by xGen, followed by sequencing on the Illumina HiSeq 2500 platform using the same protocol previously described in detail (48, 49). In summary, the sequencing coverage depth was sufficient to provide >20x haploid depth of over 85% of targeted bases in 96% of samples, with ~80x mean haploid read depth of targeted bases. Further, the reads generated for all samples (FASTQ files) were aligned to genome reference (GRCh38) using BWA-mem (50). The duplicate reads were identified and flagged using Picard MarkDuplicates tool for exclusion in later analysis (51). The variants were called using Genome Analysis Toolkit (GATK) (52, 53). The INDEL-realigned and duplicate-marked reads were processed using GATK HaplotypeCaller to identify variations from genome reference generating genomic VCF files (gVCF). Further, both single-nucleotide variants (SNVs) and indels were identified using GATK's GenotypeGVCFs after genotyping each sample and a training set consisting of 50 randomly selected samples resulting in single-sample VCF files. The joint calling was done in batches of 200 single-sample gVCFs to create pVCF files and all pVCF files generated from joint calling were merged. This process was repeated for both datasets. Further, quality control steps were applied by filtering variant SNP sites for QualityByDepth (QD) score <3 and depth <7, and indels for QD <5 and depth <10. SNP sites and indel sites that don't carry an alternate Allele Balance (AB) ≥ 15% and AB ≥ 20%, respectively, in at least one sample were filtered out. Further, markers with a call rate <90% and samples with the call rate <90% were removed. Related samples up to a 3rd degree (IBD ≥ 0.125) were removed before running the association.

### Rare Variant Gene-Based Association Test

All variants below 5% MAF were considered rare and they were binned in genes using BioBin v2.3.0 (19) (https://ritchielab.org/software/biobin-download). BioBin is a software that can collapse variants into biologically-informed bins, such as genes or pathways, and perform rare variant burden tests. BioBin uses a database called Library of Knowledge Integration (LOKI) which integrates knowledge from various disparate data sources about genomic locations of SNPs and genes, as well as known relationships among genes and proteins such as interaction pairs, pathways and ontological categories (19). The gene annotations in LOKI are derived from Entrez Gene (54). BioBin uses these annotations from LOKI as bin regions to bin the variants. After creating the bins, the variants were weighed using Madsen and Browning weights (55). All variants with MAF <5% were binned and genes with <20 variants were filtered out. SKAT-O was run using the R package (22). Additionally, age, BMI and first four principal components were used as covariates. The QQ-plot using gene based SKAT-O *p*-values is provided in Supplementary Figure 1. The *p*-values from association tests were adjusted for multiple testing using Bonferroni correction and any gene with *p*-value <5% was considered significant.

### Rare Variant Pathway-Based Association Test

Rare variants were binned into gene pathways to test the association of pathways to endometrial cancer. LOKI is integrated with KEGG pathway information (56). The version of LOKI used in this study integrated the latest data on 15 April 2017 using the KEGG API. The rare variants were binned into 302 pathways and weighed using Madsen and Browning weights (55). Associations were tested using SKAT-O, adjusting for age, BMI and first four principal components. The QQ-plot using pathway based SKAT-O *p*-values is provided in Supplementary Figure 2. The SKAT-O *p*-values were adjusted for multiple testing using Bonferroni correction and any *p*-value <5% was considered significant.

### Survival Analysis

Survival analysis was run using Cox regression adjusting for age and BMI, using the following model:

$$\begin{array}{r} \text{Survival }\left(\text{months},\text{alive}\right)\;\sim \text{x}+\text{age}+\text{BMI} \end{array}$$

Where “x” is the input from BioBin phe-bins output file which contains Madsen-browning weighted rare variant counts for each patient and gene. The “months” were measured as the number of months from birth to death for dead patients and the number of months from birth to the last follow-up recorded in the EHR for patients alive.

## Data Availability

The raw data supporting the conclusions of this manuscript will be made available by the authors to any qualified researcher subject to a data use agreement.

## Ethics Statement

The DisocvEHR study cohort is derived from individuals who consented to participate in Geisinger's MyCode Community Health Initiative as described previously (1, 57). Additionally, IRB approval was obtained for this work (IRB- 2016-0119).

## Author Contributions

MS, RG, and DK designed and conceived the project. MS, VD, and JM carried out the methodology and implementation. DK and RG helped to supervise the project. MS wrote the paper. All authors revised the manuscript and approved the final version prior to submission.

### Conflict of Interest Statement

The authors declare that the research was conducted in the absence of any commercial or financial relationships that could be construed as a potential conflict of interest.
