# Supplementary material for: Exome-Wide Rare Variant Analysis From the DiscovEHR Study Identifies Novel Candidate Predisposition Genes for Endometrial Cancer
Source: Front Oncol. 2019 Jul 5;9:574. doi: 10.3389/fonc.2019.00574 (PMC6626914; doi:10.3389/fonc.2019.00574)
Supplement: Supplementary file 2 [file Data_Sheet_2.docx]

Supplementary Figure 1. QQ-plot for gene based rare variant analysis using SKAT-O

b.

a.


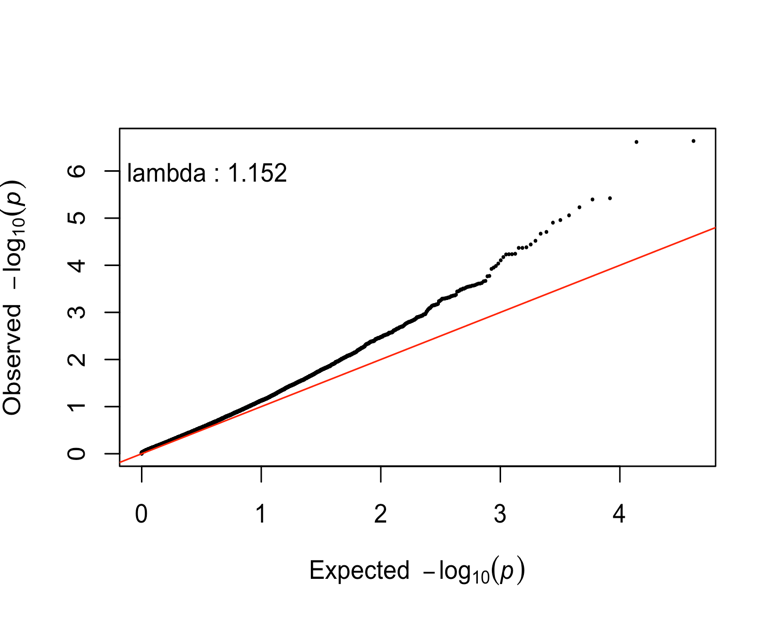

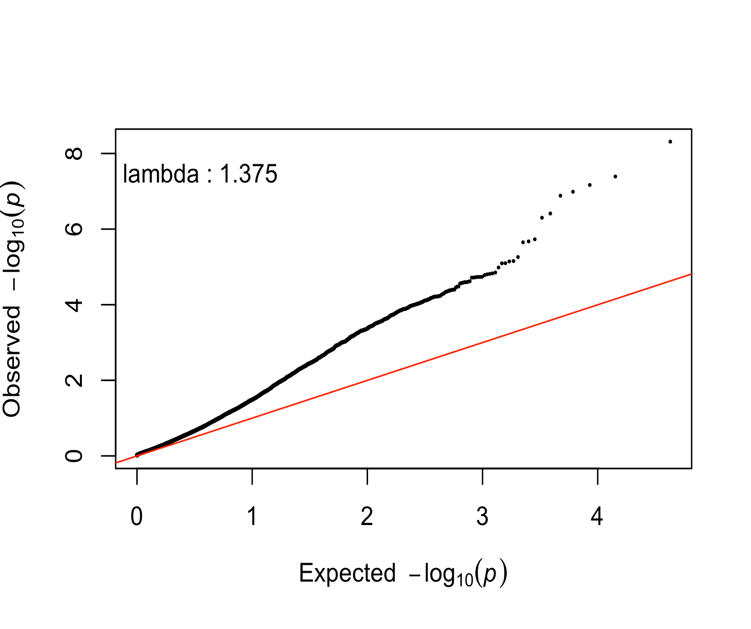


a. QQ-plot from discovery analysis. The genomic inflation rate (λ) was 1.375. The p-values show noticeable inflation, especially when significance level is smaller. The gene-based tests are known to have higher inflation rate which could be due to interpopulation distribution of rare variants or joint allelic distribution in two populations (1).

b. QQ-plot from replication analysis. The genomic inflation rate (λ) was 1.152.

Supplementary Figure 2. QQ-plot for pathway based rare variant analysis using SKAT-O


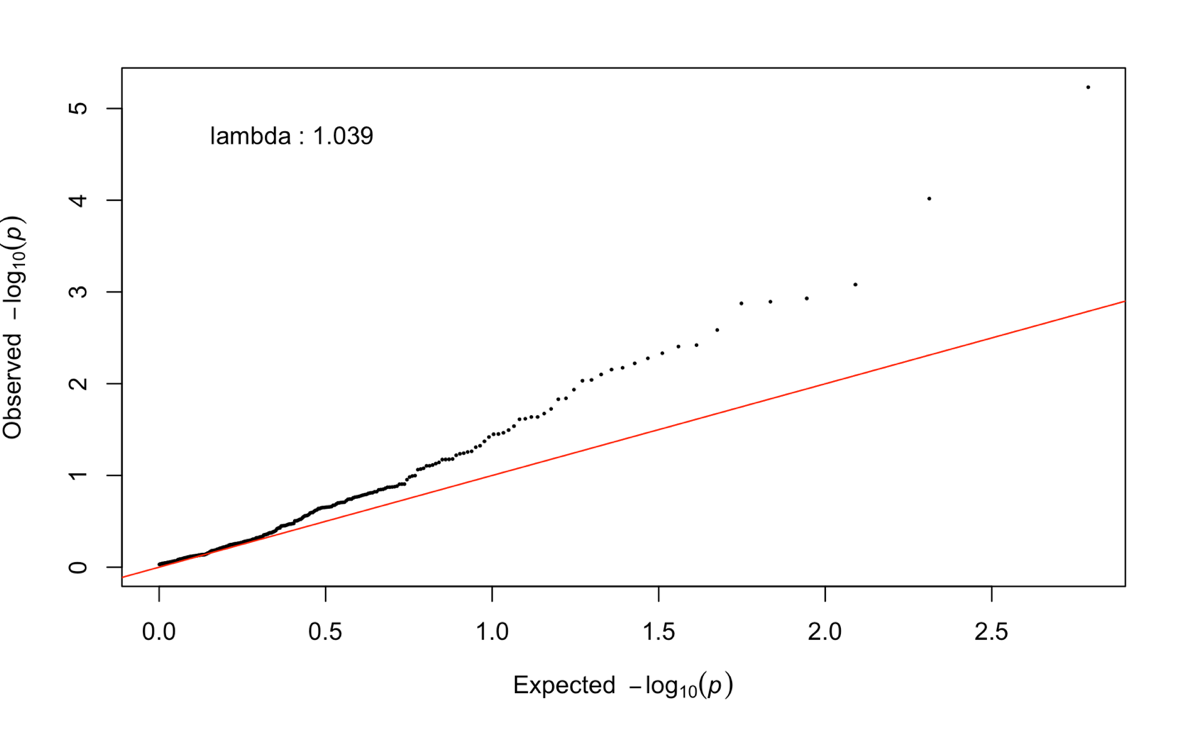


The genomic inflation rate (λ) for pathway (KEGG) based rare variant analysis was 1.039.

The source code, software information and commands used in this can be found at

https://github.com/dokyoonkim99/EMCA_rare_variant

1. Zawistowski M, Reppell M, Wegmann D, St Jean PL, Ehm MG, Nelson MR, et al. Analysis of rare variant population structure in Europeans explains differential stratification of gene-based tests. *Eur J Hum Genet* (2014) 22(9):1137-44. Epub 2014/01/08. doi: 10.1038/ejhg.2013.297. PubMed PMID: 24398795.
